# Relationship Between Metabolic Dysfunction-Associated Steatotic Liver Disease and Lipoprotein (a) and Other Biomarkers

**DOI:** 10.7759/cureus.63019

**Published:** 2024-06-24

**Authors:** Felipe A. Muñoz Rossi, Juanita Salazar Agudelo, Néstor Israel Quinapanta Castro, Sofía Z. Mosquera, Maria Clara Mejia Fajardo, Edgar Dario Mosquera López, Jonathan Dany Espitia Olarte, Maria Andrea Figueroa Medina, Edwin Zuniga Simancas, Luis Fernando Saldarriaga Osuna

**Affiliations:** 1 Internal Medicine, National University of Colombia, Bogota, COL; 2 Emergency Medicine, CES Clinic, Medellin, COL; 3 Research and Biostatistics, Regional Autonomous University of the Andes, Ambato, ECU; 4 General Medicine, Cooperative University of Colombia, Medellín, COL; 5 General Medicine, Universidad Central del Ecuador, Quito, ECU; 6 General Medicine, Libre University of Colombia, Barranquilla, COL; 7 Internal Medicine, Sinu University/General Clinic of the Caribbean/Blas de Lezo Clinic/Cardiovascular and Metabolic Center, Cartagena, COL; 8 General Physician, National University of Colombia, Bogota, COL

**Keywords:** non-alcoholic fatty liver disease, gamma glutamyl transferase (ggt), nash and steatosis, high-density lipoproteins (hdl-c), lipoprotein (a)

## Abstract

Background: Metabolic dysfunction-associated steatotic liver disease (MASLD) primarily affects the adult population and is closely related to obesity. The most severe form of MASLD, metabolic dysfunction-associated steatohepatitis (MASH), can progress to liver fibrosis. While lipoprotein(a) (Lp(a)) is known to be associated with cardiovascular disease, its relationship with MASLD remains unclear. This study aims to determine the prevalence of MASLD in ambulatory patients and to explore the association between Lp(a) levels and advanced liver damage.

Methods: This retrospective cross-sectional study included 130 patients older than 18 years seen in a healthcare center in Medellin, Colombia, between April 2023 and May 2024. Sociodemographic, clinical, and specific biomarker data were collected. Patients with cirrhosis, previous liver disease, frequent alcohol consumption, cancer, and other severe conditions were excluded. Continuous variables were analyzed using Student's t-tests or Mann-Whitney tests according to their distribution, and categorical variables were analyzed using contingency tables and chi-square tests.

Results: Of the 130 patients, 57.9% (n=73) had MASLD, with a higher prevalence in patients with obesity (80%, n=32). Lp(a) levels were abnormally high in 43.1% (n=31) of patients; however, a weak but significant inverse correlation was found between Lp(a) levels and the Fibrosis-4 (FIB-4) score, which is used to assess the severity of liver fibrosis. Patients with MASLD had significantly lower high-density lipoprotein (HDL) and vitamin D levels, and higher levels of gamma-glutamyl transferase (GGT).

Conclusions: This study highlights the significant prevalence of MASLD in outpatients and its relationship with various biomarkers, including Lp(a), HDL, vitamin D, and GGT. Although the findings suggest a possible utility of Lp(a) as a biomarker in MASLD, longitudinal studies are needed to confirm these associations and clarify their role in liver disease progression. The study's limitations include its cross-sectional nature and potential selection bias, indicating the need for further research to validate these results.

## Introduction

The prevalence of metabolic dysfunction-associated steatotic liver disease (MASLD) in the adult population is 30% [1], making it a significant public health problem. There is a correlation between the increased incidence of MASLD and the increased prevalence of obesity [2].

MASLD is diagnosed when imaging or a biopsy shows steatosis in the liver and at least one of the following conditions is present: body mass index (BMI) ≥ 25 kg/m² (≥23 kg/m² in Asians) or waist circumference >94 cm in men and >80 cm in women, adjusted for ethnicity; fasting serum glucose ≥ 100 mg/dL; two-hour postload glucose level ≥ 140 mg/dL; HbA1c ≥ 5.7% or specific pharmacological treatment; blood pressure ≥ 130/85 mmHg or specific pharmacological treatment; plasma triglycerides ≥ 150 mg/dL or specific pharmacological treatment; plasma high-density lipoprotein (HDL) cholesterol < 40 mg/dL in men and < 50 mg/dL in women [2].

On the other hand, metabolic dysfunction-associated steatohepatitis (MASH) is the inflammatory and most severe form of MASLD. It is defined by the presence of lobular inflammation and edema at the level of hepatocytes, which may progress to fibrosis. These symptoms may be present in up to 7% of MASLD patients without indication for liver biopsy [3] and in up to 63% of patients undergoing liver biopsy [4].

MASLD affects other organs and systems beyond the liver, such as the cardiovascular and endocrine systems; therefore, it is essential to manage this disease comprehensively to avoid long-term health consequences for patients. Lipoprotein(a) (Lp(a)) is considered a causal risk factor for cardiovascular atherosclerotic disease, independent of other conventional risk factors, and is also independently associated with an increased risk of aortic stenosis progression [5-7]. The significance of Lp(a) changes in patients with MASLD is seldom investigated beyond their relationship to cardiovascular risk, as evidenced by the few controversial studies on the relationship between Lp(a) concentration and other metabolic abnormalities [8]. For instance, a Korean study involving 22,534 participants indicates a higher risk of developing MASLD in patients with low Lp(a) and high insulin resistance (IR) [9]. Similarly, the Jung et al. study found, among a sample of 3030 people with MASLD, an inverse correlation of Lp(a) to the presence of fatty liver [10].

The current findings have not provided definitive conclusions on the implications of Lp(a) alteration in MASLD. Therefore, the main objective of this study is to determine the prevalence of MASLD in ambulatory patients and, in turn, determine whether Lp(a) levels are associated with advanced liver damage. This analysis supports the hypothesis that Lp(a) assessment may be an early biomarker in patients with MASLD to predict liver fibrosis.

## Materials and methods

This retrospective cross-sectional study was conducted in a private medical care center in Medellin, Colombia. One hundred thirty clinical histories were collected from patients over 18 admitted as outpatients for general follow-up based on their age group between April 2023 and May 2024. To achieve the study's objectives, sociodemographic, clinical, and specific biomarker data were collected to determine the study's objective and to create the database.

Inclusion criteria included patients over 18 years of age admitted on an outpatient basis for general assessment, defined as a complete clinical evaluation including clinical history and relevant laboratory tests according to their age group in previously asymptomatic patients, with abdominal ultrasound during the observation period.

Exclusion criteria included patients with cirrhosis, previous liver diseases such as viral hepatitis, autoimmune hepatitis, herbal and/or drug-induced liver damage, acute or chronic pancreatitis, cancer of any type and/or stage, acute and chronic renal disease, pregnant patients, frequent alcohol consumption defined as more than 20 g/day, and use of psychoactive substances.

A sample size was calculated for a mean difference between two independent groups, accepting an alpha risk of 0.05 and a beta risk of 0.2 in a bilateral contrast. For a statistical power of 80%, 126 subjects are required. The proportion in the reference group is assumed to be 0.5.

Categorical variables were handled in frequencies and percentages using contingency tables. In contrast, continuous variables were shown using measures of central tendency such as the median or mean according to their normal distribution, determined by the Kolmogorov-Smirnov test.

The means of continuous quantitative variables were compared with Student's t-tests if they had a normal distribution; if not, a nonparametric test, specifically the Mann-Whitney test, was performed if pertinent. On the other hand, the comparison of qualitative variables was carried out using the chi-square test, first performing a univariate analysis, and according to the results, comparison analyses were performed on those with a p-value < 0.05.

Each biomarker was treated quantitatively and qualitatively. The clinical laboratory of the institutional center that extracted the data determined the reference ranges, and all analyses were carried out using the IBM SPSS Statistics for Windows, Version 27 (Released 2020; IBM Corp., Armonk, New York), R Studio, and Jamovi, establishing statistical significance with a level of p < 0.05.

Ethical considerations

Considering the study's retrospective nature, this research is considered low-risk. No intervention was performed that could modify the participants' behaviors and treatments, so informed consent was not required. The confidentiality of the health information was guaranteed without mentioning any personal data or information about the medical personnel involved. Approval was requested from the corresponding ethics committee of the health institution for this study, adhering to the guidelines of good clinical practice.

## Results

The study included 130 patients admitted to the internal medicine outpatient program for a general examination. Tables 1, 2 summarize the main clinical and biochemical characteristics of the participants.

**Table 1:** Demographic characteristics BMI: body mass index, Lp(a): lipoprotein(a), HbA1c: hemoglobin A1c, LDL: low-density lipoprotein, HDL: high-density lipoprotein, GGT: gamma-glutamyl transferase, AST: aspartate aminotransferase, ALT: alanine aminotransferase.

Variable (n =130)	Mean	SD	95% CI
Age	50	12.6	47.8–52.2
Height	165.7	11.22	163.7–167.7
Weight	77.27	15.89	74.49–80.05
BMI	27.7	4.58	26.9–28.5
Lp(a)	81.66		60.79–102.53
HbA1c (median)	5.4		4.83–12.29
Cholesterol	206	42.4	198–213
LDL	129	38.2	123–136
HDL	49.5	14.3	46.9–52.0
Triglycerides	143	77.6	129–156
GGT	36.4	31	30.1–42.8
AST	26.1	11.4	24.1–28.2
ALT	30.7	19.4	27.2–34.3
Vitamin D	30.3	9.91	27.8–32.8

**Table 2:** Comorbidities MASLD: metabolic dysfunction-associated steatotic liver disease.

Comorbidity	No (%)	Yes (%)
Arterial hypertension	92 (71.3%)	37 (28.7%)
Dyslipidemia	71 (55.0%)	58 (45.0%)
Diabetes mellitus	123 (95.3%)	6 (4.7%)
MASLD	53 (42.1%)	73 (57.9%)

**Figure 1:**
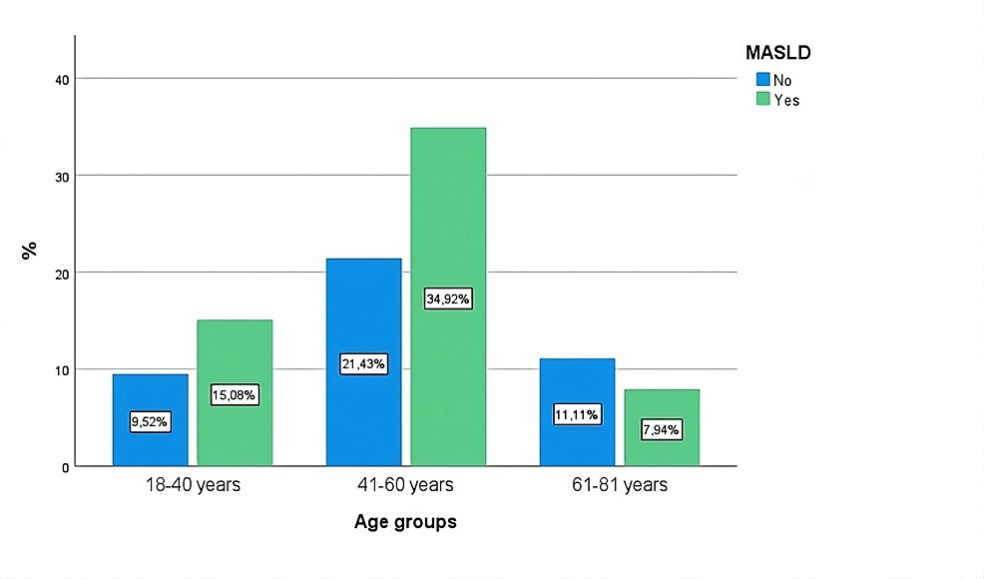
MASLD bar chart by age group

In the present sample, the majority were women, comprising 51.5% (n = 67), compared to 48.5% (n = 63) of men. The mean age was 50 years (95% CI: 47.8-52.2), and most subjects were 41-60 years old, making up 54.6% (n = 71) of the sample. Within this range, 34.9% (n = 44) presented with MASLD (Figure 1).

Characteristics of patients with MASLD

The prevalence of patients with MASLD was 57.9% (n = 73; 95% CI = 49.2%-66.7%), with steatohepatitis associated with metabolic dysfunction in 38.4% (n = 28), and a median BMI of 27.36 (95% CI 27.1-29.3).

**Figure 2:**
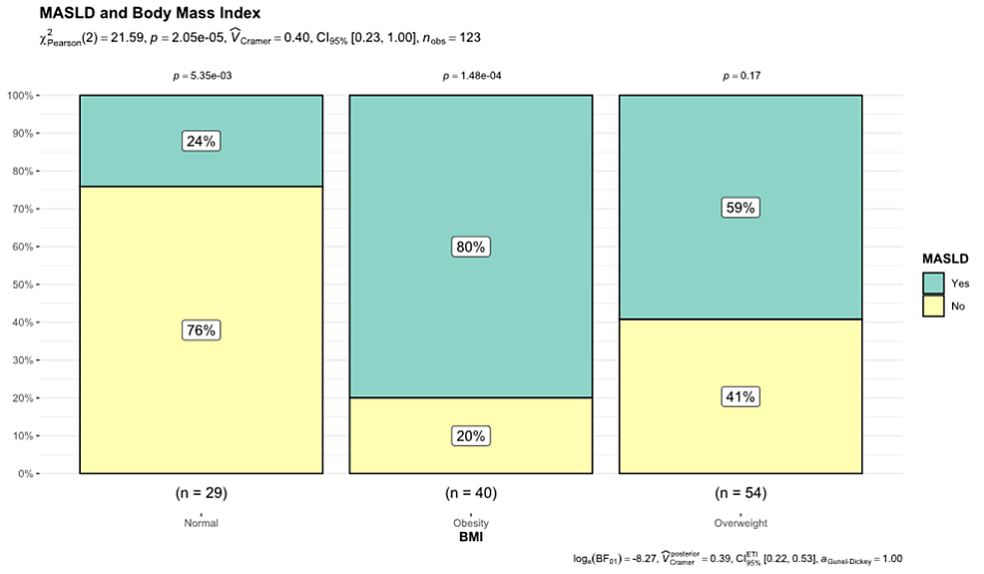
Proportion of MASLD and weight categories MASLD: metabolic dysfunction-associated steatotic liver disease.

Among the patients with obesity, 80% (n = 32) presented with MASLD, while 59% of the overweight group and only 24% of those with a standard BMI were affected. This trend suggests that BMI is a significant risk factor for MASLD, demonstrating a clear and significant relationship between higher BMI and higher prevalence of MASLD (Figure 2).

Consistent with the findings of the present study, a 43.1% (n = 31) prevalence of abnormally elevated Lp(a) levels was found. Furthermore, we observed abnormal Lp(a) levels in 44.7% (n = 17) of MASLD patients. Therefore, the majority of patients with MASLD did not show elevated Lp(a) levels.

**Figure 3:**
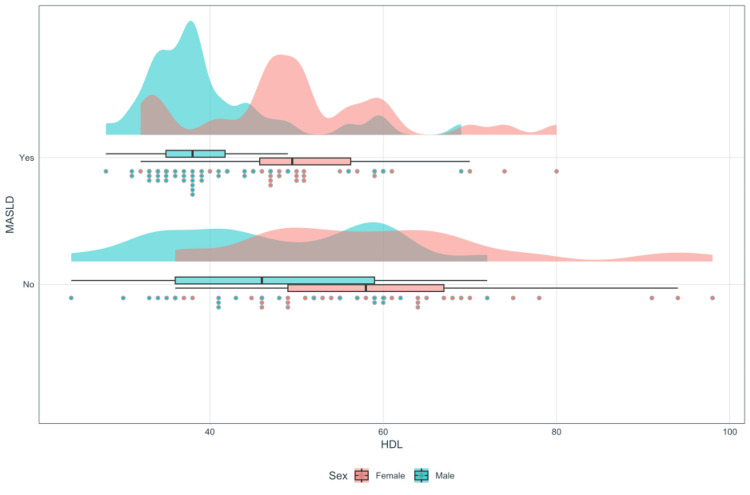
Raincloud MASLD and HDL MASLD: metabolic dysfunction-associated steatotic liver disease, HDL: high-density lipoprotein.

The mean HDL in patients with MASLD was 44.6, compared to 54.7 in those without MASLD, with a statistically significant between-group difference (p = 0.000). When grouped by gender, this difference is primarily evident in male patients (Figure 3 and Table 3).

**Table 3:** Description of MASLD and HDL classified by gender. The CI of the mean assumes that sample means follow a t-distribution with N - 1 degrees of freedom. MASLD: metabolic dysfunction-associated steatotic liver disease, HDL: high-density lipoprotein.

					95% Confidence Interval		
MASLD		Gender	N	Mean	Lower	Upper	Median
HDL	No	Male	21	47.0	41.3	52.8	46.0
		Female	33	59.6	54.2	65.1	58.0
	Yes	Male	39	40.0	37.2	42.7	38.0
		Female	32	50.2	46.1	54.3	49.5

Concerning the atherogenic TC/HDL index, it was found that patients with MASLD presented a higher value, with a median of 4.66 compared to 3.83 in patients without the disease. The mean TC/HDL index was 4.85 (95% CI = 4.55-5.15) in patients with MASLD and 4.01 (95% CI = 3.64-4.37) in those without MASLD, demonstrating a significant difference as indicated by the Mann-Whitney statistic (p < 0.001) (Table 4).

**Figure 4:**
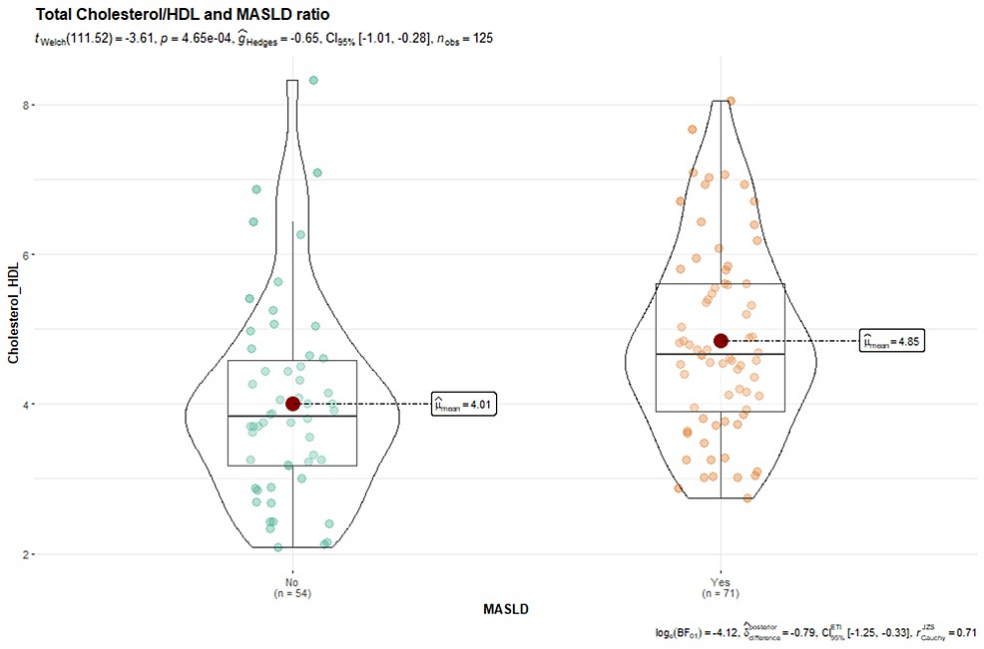
Box plot: cholesterol/HDL and MASLD MASLD: metabolic dysfunction-associated steatotic liver disease, HDL: high-density lipoprotein.

**Table 4:** Comparison of the atherogenic index total cholesterol/HDL between groups (MASLD) Independent sample t-test MASLD: metabolic dysfunction-associated steatotic liver disease, HDL: high-density lipoprotein.

		Statistic	df		p
Cholesterol/HDL	Student's t	-3.63		123	< .001
	Mann-Whitney U	1159			< .001

The subjects in the MASLD group had an average vitamin D level of 27.75 ng/mL, whereas those without MASLD had an average of 33.29 ng/mL. Consequently, these data show that people without hepatic steatosis may have higher vitamin D levels than those diagnosed with MASLD.

**Figure 5:**
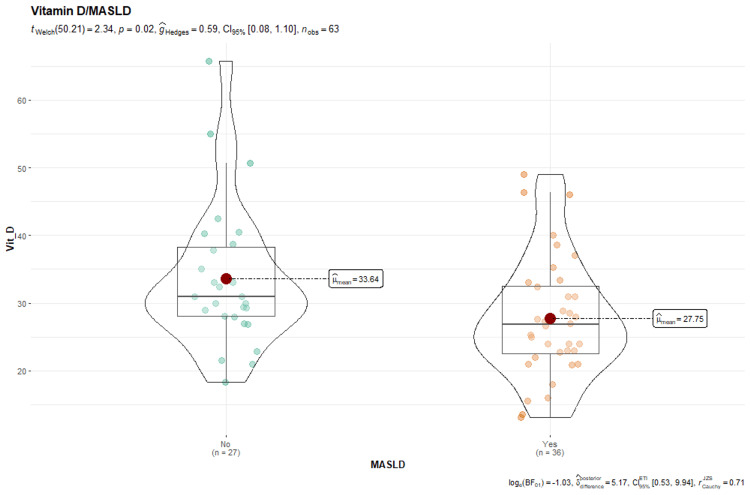
Box plot: vitamin D/MASLD MASLD: metabolic dysfunction-associated steatotic liver disease.

The results of Student's t-test reveal a significant difference between the groups (Student's t-test (2.4), p = 0.0019). Furthermore, the effect size using the Hedges estimator (^Hedges = 0.58) suggests a difference in vitamin D levels between the two groups. Based on the presented data, subjects with MASLD tend to have lower vitamin D levels than those without the disease; this finding may indicate a probable relationship between low vitamin D levels and the existence of fatty liver disease (Figure 5).

**Figure 6:**
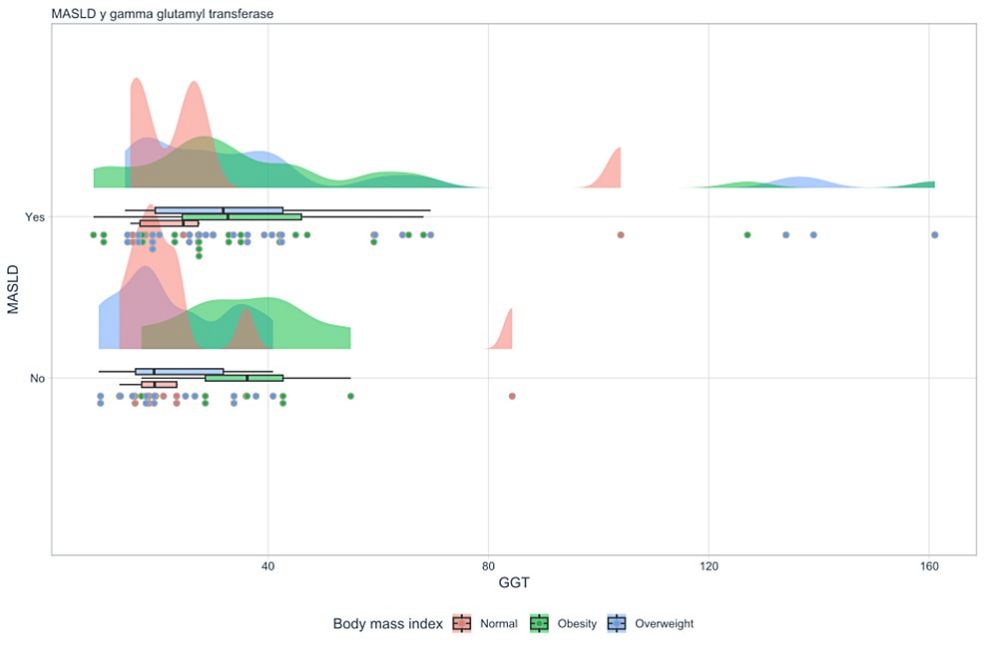
Raincloud MASLD and GGT MASLD: metabolic dysfunction-associated steatotic liver disease, GGT: gamma-glutamyl transferase.

In the scope of the present investigation, it was shown that there is a clear correlation between gamma-glutamyl transferase (GGT) levels and the existence of MASLD. The data reveal that individuals with MASLD have significantly higher levels of GGT compared to those without the disease, with a mean of 44.4 (95% CI = 34.9-53.8) in patients with MASLD and a mean of 26.9 (95% CI = 21.4-32.4) in patients without MASLD, according to a nonparametric test (Mann-Whitney U = 694, p = 0.018) (Table 5). Additionally, classifying this correlation according to BMI categories (Figure 6) demonstrates that people with MASLD have significantly higher and more variable GGT levels, especially those who are overweight and obese, compared to people with a normal BMI. This situation is particularly evident for overweight individuals.

**Table 5:** Results of normality tests for GGT levels Additional results provided by more tests. GGT: gamma-glutamyl transferase.

Test		p
GGT	Shapiro-Wilk	<0.001
	Kolmogorov-Smirnov	0.001
	Anderson-Darling	<0.001

According to this pattern, having a higher BMI increases the likelihood of developing MASLD. It is also associated with increased hepatic stress, as reflected in GGT levels. In contrast, when MASLD is absent, GGT levels are lower and less variable, although they still show a minor tendency to increase with BMI.

Correlation analysis between Lp(a) and the FIB-4 score

**Figure 7:**
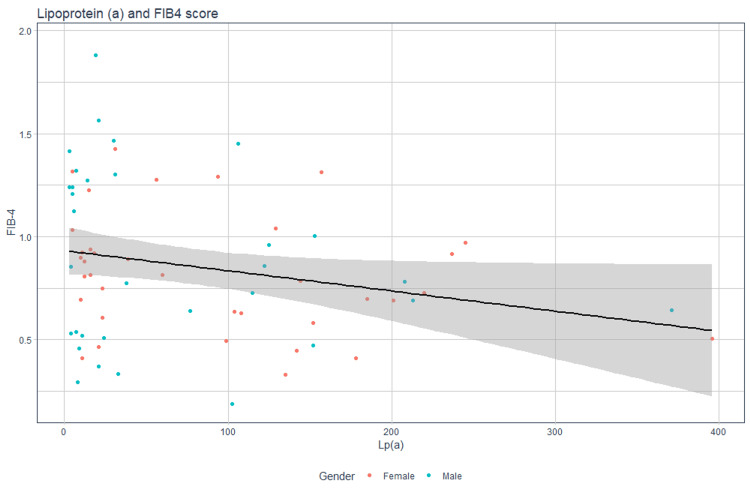
Scatterplot: correlation between Lp(a) and FIB-4 score Lp(a): lipoprotein(a), FIB: fibrosis.

In the present investigation, a weak but significant negative correlation was evidenced between the FIB-4 score, which establishes the severity of MASLD, and Lp(a) levels. Consequently, we can propose that the lower the Lp(a) levels, the higher the FIB-4 score, with a statistically significant association (r = -0.241, p = 0.048) (Figure 7, Table 6).

**Table 6:** Correlation between Lp(a) and FIB-4 score Lp(a): lipoprotein(a), FIB: fibrosis.

Measure	Value
Pearson's r	-0.241
p-value	0.048

Although weak, this inverse relationship could be considered a factor to be evaluated in the context of the integral management of MASLD and its complications, such as progression to fibrosis. 

## Discussion

The aim of this study was to explore the probable relationship between lipid profiles, particularly Lp(a), and hepatic steatosis related to metabolic dysfunction. Our findings significantly contribute to therapeutic implications, providing a solid framework for future research.

A predominance of men is evident among patients with MASLD, with 33.3% being male compared to 24.8% female. This may be related to a higher prevalence of overweight/obesity among men (30%) compared to women (22%), which could be a confounding factor when analyzing gender ratios.

In our sample, the prevalence of MASLD was 57.9% (n = 73; 95% CI = 49.2%-66.7%), with a mean age of 48.7 years (95% CI = 46.1-51.3) and a mean BMI of 29.6 (95% CI = 28.6-30.6). Regional studies suggest that the proportion may be as high as 46.04% (95% CI = 40.55-51.60) [11]. The overall global prevalence of MASLD is 25% (95% CI = 22.1-28.65) [3]. An investigation conducted in Brazil found the frequency to be 35.2% [12], while locally, Perez et al. found the prevalence in Colombia to be approximately 27% (95% CI = 21.38-32.39) [13].

Regarding the lipid profile, 43.1% (n = 31) of the sample had higher Lp(a) levels. However, only 23.6% of patients with hepatic steatosis showed abnormally high levels of this lipoprotein. Additionally, HDL levels were significantly lower in patients with MASLD, with a mean of 44.6 (95% CI = 42.0-47.2) compared to subjects without MASLD, who had a mean of 54.7 (95% CI = 50.5-59.0), showing a statistically significant difference (p = 0.000).

Other studies have found similar results, indicating an inverse correlation between HDL cholesterol levels and the severity of hepatic steatosis in MASLD. Bril et al. found that patients with MASLD had mean HDL levels of 43 mg/dL, compared to 48 mg/dL in those without MASLD, with a statistically significant difference (p = 0.05) [14]. Other investigations also indicated that individuals with hepatic steatosis, a condition linked with metabolic dysfunction, had substantially lower HDL cholesterol levels than controls without the condition [15].

Moreover, the atherogenic indicator, the ratio of total cholesterol to HDL levels, was more significant in participants with MASLD than in those without the condition [16]. Our research also reported similar, statistically significant results, indicating that patients with the highest atherogenic index had MASLD.

An additional observation was the decreased mean vitamin D levels in patients with MASLD, with a value of 27.75 ng/mL (95% CI = 24.8-30.7), showing a statistically significant difference (p = 0.0019). This demonstrates that low vitamin D concentrations could be related to the occurrence of MASLD, which is consistent with past studies associating vitamin D deficiency with metabolic diseases.

Kumar et al., in a cross-sectional study, found a correlation between vitamin D deficiency and patients with MASLD with statistical significance (p = 0.04) [17]. Additionally, a systematic review highlighted the possibility that vitamin D could improve some inflammatory mediators in MASH, although more randomized controlled trials (RCTs) are needed to determine the fundamental role of vitamin D in hepatic steatotic disease associated with metabolic dysfunction [18].

This study provides evidence of a correlation between GGT levels and the presence of MASLD. Similar findings in other studies conclude that elevated GGT levels may increase the risk of MASLD [19]. Therefore, GGT could be a useful clinical biomarker for identifying patients at increased risk of disease complications.

Finally, the correlation analysis showed a weak but significant negative relationship between Lp(a) levels and the FIB-4 score (r = -0.241, p = 0.048). This suggests that lower levels of Lp(a) are associated with a higher FIB-4 score and, thus, with greater severity of hepatic fibrosis. Although this correlation is weak, its statistical significance indicates that Lp(a) may be a crucial factor in assessing the risk of progression in patients with MASLD.

While consideration is given to the possibility of confounding factors that may influence the relationship between Lp(a) and the FIB-4 score, a hypothesis is formed that can be used in further research to reduce the probability of Type I error. Several studies have found similar findings: Meroni et al. provided evidence that low Lp(a) levels may serve as a non-invasive biomarker for predicting advanced fibrosis in patients with MASLD [20]. Similarly, a Korean study found an inverse relationship between Lp(a) levels and the severity of MASLD [21].

However, our study has certain limitations. First, given the cross-sectional nature of the study, we cannot discern a cause-and-effect relationship between the variables under consideration. While strong links have been observed, caution should be exercised when making inferences regarding the cause or impact of Lp(a), HDL, or vitamin D levels on MASLD.

To counteract selection bias, we took several measures. For instance, a pilot test of the selected variables was conducted with 30 participants, and data were gathered. Based on the findings, modifications were made to ensure complete and accurate data capture, thereby evaluating the quality of the information.

The external validity of the data reported is further limited as the sample, albeit significant, is confined to patients who attended internal medicine outpatients, which may not truly represent the wider population. Furthermore, longitudinal data are unavailable, given the absence of prospective follow-up, which would better help understand the temporal development of Lp(a) levels and other biomarkers associated with MASLD.

## Conclusions

These data imply that Lp(a) may have a relevant role in modulating the risk of hepatic fibrosis, generating hypotheses about the associated pathophysiological processes. Furthermore, we highlight the importance of this parameter as a possible biomarker for the risk of progression in subjects with hepatic steatosis. This might have substantial therapeutic implications for reducing liver fibrosis and treating the illness.

Moreover, correlations were observed between MASLD and other biomarkers, such as vitamin D deficiency, demonstrated in similar studies. Similarly, GGT levels were significantly elevated in individuals with MASLD. Further research is needed to corroborate these findings and the possible pathophysiological mechanisms responsible for this probable link.
